# Mir-29 Repression in Bladder Outlet Obstruction Contributes to Matrix Remodeling and Altered Stiffness

**DOI:** 10.1371/journal.pone.0082308

**Published:** 2013-12-10

**Authors:** Mari Ekman, Anirban Bhattachariya, Diana Dahan, Bengt Uvelius, Sebastian Albinsson, Karl Swärd

**Affiliations:** 1 Department of Experimental Medical Science, Lund University, Lund, Sweden; 2 Department of Urology, Lund University, Lund, Sweden; 3 Department of Biology, Lund University, Lund, Sweden; National Institutes of Health, United States of America

## Abstract

Recent work has uncovered a role of the microRNA (miRNA) miR-29 in remodeling of the extracellular matrix. Partial bladder outlet obstruction is a prevalent condition in older men with prostate enlargement that leads to matrix synthesis in the lower urinary tract and increases bladder stiffness. Here we tested the hypothesis that miR-29 is repressed in the bladder in outlet obstruction and that this has an impact on protein synthesis and matrix remodeling leading to increased bladder stiffness. c-Myc, NF-κB and SMAD3, all of which repress miR-29, were activated in the rat detrusor following partial bladder outlet obstruction but at different times. c-Myc and NF-κB activation occurred early after obstruction, and SMAD3 phosphorylation increased later, with a significant elevation at 6 weeks. c-Myc, NF-κB and SMAD3 activation, respectively, correlated with repression of miR-29b and miR-29c at 10 days of obstruction and with repression of miR-29c at 6 weeks. An mRNA microarray analysis showed that the reduction of miR-29 following outlet obstruction was associated with increased levels of miR-29 target mRNAs, including mRNAs for tropoelastin, the matricellular protein Sparc and collagen IV. Outlet obstruction increased protein levels of eight out of eight examined miR-29 targets, including tropoelastin and Sparc. Transfection of human bladder smooth muscle cells with antimiR-29c and miR-29c mimic caused reciprocal changes in target protein levels *in vitro*. Tamoxifen inducible and smooth muscle-specific deletion of Dicer in mice reduced miR-29 expression and increased tropoelastin and the thickness of the basal lamina surrounding smooth muscle cells in the bladder. It also increased detrusor stiffness independent of outlet obstruction. Taken together, our study supports a model where the combined repressive influences of c-Myc, NF-κB and SMAD3 reduce miR-29 in bladder outlet obstruction, and where the resulting drop in miR-29 contributes to matrix remodeling and altered passive mechanical properties of the detrusor.

## Introduction

MicroRNAs (miRNAs) are small single-stranded RNAs that control protein synthesis via messenger RNA (mRNA) degradation and translational repression [1]. Individual miRNAs have hundreds of predicted targets and close to one third of all protein-coding genes and, essentially, all biological pathways are influenced by miRNAs [2]. MiRNA-based therapeutics have progressed considerably in recent years, suggesting that this novel strategy may be brought to clinical application soon [3]. Considerable work is needed to fully uncover the potential of miRNAs as therapeutic targets, and the exploration of miRNAs in urologic conditions other than cancer is in its infancy. The endonucleases Drosha and Dicer are required for miRNA maturation [4], and in recent work, we and others have taken advantage of conditional and smooth muscle-specific deletion of Dicer to probe the role of miRNAs in detrusor smooth muscle [5,6]. This leads to impairment of cholinergic neurotransmission, to a relative increase in the purinergic component of detrusor activation and to a disturbed micturition pattern. 

The miR-29 cluster has gained recognition as a modulator of extracellular matrix production [7-10]. It comprises three miRNAs (miR-29a, miR-29b, and miR-29c) derived from two independent genes [10]. Among the targets are several collagen isoforms, laminin, and elastin (Eln or tropoelastin) [11]. The extracellular matrix molecule elastin is one of the best established targets of miR-29, and its message has 14 binding sites dispersed over the coding sequence and the 3’UTR [12]. The matricellular protein Sparc has three miR-29 binding sites clustered in its proximal 3’ UTR, and, similar to elastin, this protein is effectively regulated by miR-29 *in vitro* [13]. MiR-29-mediated extracellular matrix remodeling has been demonstrated in the infarcted heart [10] and during aortic aneurysm progression [7-9], but miR-29 also plays roles in cell proliferation, muscle differentiation and apoptosis [11]. The latter effects are mediated by in part by signaling proteins and transcriptional regulators, including the ERK1/2 inhibitor Spry1 (sprouty homolog 1) [14] and the transcription factor Mybl2 (B-Myb) [15]. Studies using cultured cells support the idea that transforming growth factor-β (TGF-β), a central mediator in fibrogenesis, represses miR-29 [16]. SMAD proteins belong to a conserved family of TGF-β signal transducers that are regulated by phosphorylation [17], and the repression of miR-29 by TGF-β was shown to involve SMAD3 [16]. Additional regulatory inputs on miR-29 expression include c-Myc and NF-κB [11], and recent work has provided considerable insight into c-Myc-mediated repression, which appears to depend on a repressor complex consisting of c-Myc, histone deacetylae 3 (Hdac3) and enhancer of zeste homologue 2 (Ezh2) [18]. 

It is well established that bladder outlet obstruction, such as seen in aging men with prostate enlargement, results in substantial growth of the bladder, and we [19] and others [20-22] (reviewed by Aitken and Bagli [23]) have demonstrated that long-term obstruction leads to an increased matrix volume in the bladder and to increased detrusor stiffness. Outlet obstruction moreover increases TGF-β mRNA levels [24], and work with genetically modified mice has demonstrated a role of TGF-β receptor II (Tgfbr2) in the stiffness change that follows upon chronic obstruction [25]. We therefore hypothesized that outlet obstruction leads to SMAD3 phosphorylation repressing miR-29, and that this in turn has an impact on protein synthesis and mechanical properties of the bladder. To address this hypothesis we examined if SMAD proteins are phosphorylated and whether miR-29 is reduced in outlet obstruction. We also examined if the reduction of miR-29 correlated with altered miR-29 target mRNAs, including tropoelastin and Sparc. To address the functional impact of miR-29, we transfected a miR-29 inhibitor and mimic *in vitro* and conditionally deleted Dicer *in vivo* [5] and examined the effect of these interventions on tropoelastin expression and on tissue mechanical properties. Our studies support a model in which multiple signaling pathways converge on repression of miR-29 in outlet obstruction, facilitating matrix protein expression and leading to altered mechanical properties of the urinary bladder. 

## Materials and Methods

### Ethics statement

The Malmö/Lund animal and human ethics committees approved all procedures (M300-08, M212-13, M259-11, 2008-4) which were in conformity with international guidelines. Written informed consent was obtained for use of human bladder tissue in basic research following cystectomy.

### Surgery

Access to the urethra was gained via a midline abdominal incision in female Sprague-Dawley rats (200g) anaesthetized with 100 mg/kg ketamine (Ketalar, Parke-Davis, Barcelona, Spain) and 15 mg/kg xylazin (Rompun: Bayer AG, Leverkusen, Germany), given intramuscularly. A 1-mm Ø steel rod was positioned beside the urethra, and 4-0 Prolene was tightened around the rod and the urethra. The rod was removed, creating a partial obstruction. Sham-operated controls were treated identically but without tightening of the ligature. One group of rats was reoperated (at 6 weeks) to remove the obstruction and sacrificed 10 days later. All animals (sham, 10 days obstructed, 6 week obstructed and 10 days de-obstructed) were euthanized using CO_2_ and the bladders were weighed, separated in two longitudinal halves, and either frozen in liquid nitrogen, fixed for immunofluorescence staining or put in physiological buffer for stiffness determination. The weight of sham-operated control bladders in the array series was 80±3 mg (n=6) whereas at the weight of 10 day and 6 week obstructed bladders and bladders de-obstructed for 10 days was 401±82 (n=6), 479±94 (n=8) and 327±29 mg (n=6), respectively. Untreated control rats were used for the elastase experiment.

### Dicer KO mice

Mice with tamoxifen-inducible and smooth muscle-specific inactivation of Dicer were used at 5-10 weeks after tamoxifen or vehicle (1:10 EtOH in sunflower oil) treatment [5] (n=12+12) as indicated.

### Microarrays

Frozen bladders were homogenized using a Tissuelyser (Qiagen, Valencia, CA, USA). RNA was extracted using the miRNeasy Kit (Qiagen), followed by RNeasy MinElute Cleanup (Qiagen). Purity and concentration were determined with an ND-1000 spectrophotometer (Nanodrop Technologies Inc., Wilmington, DE, USA), and integrity was determined using a 2100 bioanalyzer (Agilent Technologies Inc., Santa Clara, CA, USA). Affymetrix RaGene-1_0-st-v1 and miRNA-3_0 arrays (Affymetrix, Santa Clara, CA, USA) were used. Basic Affymetrix chip and experimental quality analyses were performed using Expression Console software (Affymetrix) (v1.1.2). Robust multiarray analysis [26] was used for probe summarization and data normalization. Probes with signals below the median negative control were filtered. When mRNA levels from the microarrays are plotted as fold change, the comparisons are always versus the sham-operated controls. The only exception is the initial screen for miRNA-mRNA associations where the right-hand data cluster is relative to 6 weeks of obstruction. The microarray data has been deposited with Gene Expression Omnibus (accession number GSE47080, scheduled to be released on Oct 31, 2013).

### Real-time quantitative PCR to confirm reduced expression of miR-29

Total RNA was isolated using miRNeasy mini kit (Qiagen, Valencia, CA #217004) and 300ng of template RNA was reverse transcribed to cDNA using miScript II RT Kit (Qiagen, #218161) according to the manufacturer’s instructions. The relative expression of miRNAs was measured by a real time thermal cycler (StepOnePlus^TM^, Applied Biosystems) using miScript SYBR Green PCR Kit (Qiagen, # 218073) and miScript Primer Assays (for miR-29c, miR-29b, RNU6-2 and SNORD-95). The following run protocol was used: initial activation step at 95°C for 15 min followed by 3 step cycling of denaturation (at 94°C for 15s), annealing (at 55°C for 30s) and extension (at 70°C for 30s). RNU6-2 and SNORD-95 were used as endogenous controls. Eln was quantified using Quantitect (Qiagen) primer assays as described [27].

### Cell culture and transfection

Smooth muscle cells were isolated by enzymatic digestion [27] of bundles derived from healthy regions of cystectomized human bladders. For treatment with transforming growth factor β1 (TGF-β1), cells were serum starved for 24 h prior to TGF-β1 (TGF-β1: R&D Systems, Minneapolis, MN, USA; 5 ng/ml) or vehicle treatment for 48h. For miR-29c inhibitor and mimic experiments, cells were transfected with miR-29c inhibitor (Thermo Scientific, Pittsburgh, PA, USA; 20nM), mimic (Mission miRNA: Sigma-Aldrich, St. Louis, MO, USA; 100 nM) or negative control (Mission miRNA: Sigma-Aldrich, St. Louis, MO, USA; 20 and 100 nM, respectively) using Oligofectamine transfection reagent (Life Technologies). All experiments with human cells are from 3-6 independent replicates using cells from 1-2 individuals. 

### Western blotting

The same rat bladders that were used in the microarray experiments were used (sham, 10 days, 6 weeks). An independent series of sham-operated and obstructed rats (2 days, 4 days, 10 days and 6 weeks) as well as bladders from control and Dicer KO mice were also used. After removal of the bladders and snap freezing, homogenization, gel electrophoresis and transfer to nitrocellulose membranes were performed as described [5,28]. 20 µg protein was loaded per lane. Membranes were cut horizontally in 2-4 pieces and incubated with different primary antibodies. When bands were close but not overlapping, membranes were stripped using Restore PLUS Western blot stripping buffer (Thermo Scientific). Overlapping bands, such as ERK1/2 and P-ERK1/2 were assayed using different but identically loaded gels so as to avoid the risk of incomplete membrane stripping. The primary antibodies used are listed in Table 1 and were diluted as recommended by the manufacturers. Bands highlighted by arrowheads in the figures migrated at the expected molecular weight indicated on the antibody data sheet for the respective antibody. The identity of additional bands occasionally present above and below the expected molecular weight is not known. Mature elastin is, similar to collagens, a highly cross-linked and insoluble complex that cannot be separated by conventional SDS gel electrophoresis. We therefore measured the precursor protein tropoelastin. Horseradish peroxidase-conjugated secondary antibodies (1:5000 to 1:20 000 dilutions; 7074, 7076; Cell signaling) and West Femto chemiluminescence reagent (Pierce, Rockford, IL, USA) were used, and images were acquired in a LI-COR Odyssey Fc (LI-COR Biosciences, Lincoln, NE, USA). For quantification, the chemiluminescence signal within the smallest possible rectangle centered over the band was normalized to β-actin in the same lane. Ratios were subsequently normalized to the mean of the sham operated controls. 

**Table 1:** Product names, product numbers and manufacturers of primary antibodies used in the western blot experiments.

**Product name**	**Product number**	**Manufacturer**
Phosphorylated SMAD proteins (P-SMAD) P-SMAD 2/3	8828	Cell Signaling, Beverly, MA, USA
P-SMAD 1/5/8	9511	Cell Signaling
Phosphorylated extracellular-signal-regulated kinase (ERK), P-ERK 1/2	9102	Cell Signaling
ERK ½	9107	Cell Signaling
Fibrillin 1 (Fbn1)	AV37969	Sigma-Aldrich, St. Louis, MO, USA
Laminin γ-1 (Lamc1)	sc-5584	Santa Cruz Biotechnology, Inc., Dallas, TX, USA
Intergrin β1 (Itgb1)	82373	Thermo Scientific, Pittsburgh, PA, USA
V-myb myeloblastosis viral oncogene homolog (avian)-like 2 (Mybl2; also known as B-Myb)	SAB2500168	Sigma-Aldrich
FBJ murine osteosarcoma viral oncogene homolog (Fos; also known as c-Fos)	4384	Cell Signaling
Elastin (tropoelastin, Eln)	sc-374638	Santa Cruz Biotechnology, Inc.
Eln	MAB2503	Millipore, Billerica, MA, USA
Secreted protein, acidic, cysteine-rich (osteonectin) (Sparc)	GTX19528	GeneTex, San Antonio, TX, USA
Sprouty homolog 1, antagonist of FGF signalling (Spry1)	SAB2102291	Sigma-Aldrich
Glyceraldehyde-3-phosphate dehydrogenase (Gapdh)	MAB374	Millipore
Caveolin 1, Caveola protein, 22kDa (Cav1)	3267	Cell Signaling
Phsopho NF-κB p105	4806	Cell Signaling
Phospho-NF-κB p65	3033	Cell Signaling
IκB-α	9242	Cell Signaling
c-Myc (Myc)	5605	Cell Signaling
Enhancer of Zeste homolog 2 (Ezh2)	4905	Cell Signaling

### Transcription factor binding site analysis

The role of transcription factors responsible for altered gene expression in a microarray experiment can be indirectly gauged using transcription factor binding site (TFBS) analysis. This method uses TFBS/promoter databases generated by a Systematic Motif Analysis Retrieval Tool and identifies TFBS (motifs) that are significantly enriched by a re-sampling and ranking procedure [29]. This yields a *p* value for the probability to acquire the level of enrichment of certain motifs by chance. TFBS analysis was used to identify significantly enriched motifs in the 10 days obstruction vs. sham data set. The cut-off criteria applied for the input gene list were q=0 and either >1.2-fold or <0.8-fold change, respectively.

### Immunofluorescence

Bladders were fixed, sectioned and mounted as described [28]. Slides with 10-µm equatorial sections were incubated overnight (4°C) with rabbit collagen IV antibody (ab6586; Abcam, Cambridge, MA, USA) in phosphate-buffered saline (PBS; pH 7.2) containing 0.25% bovine albumin and 0.25% Triton X-100. After rinsing and incubation with anti-rabbit immunoglobulin G coupled to Alexa fluor 594 nitrogen (Molecular Probes, Eugene, OR, USA), micrographs were acquired in a fluorescence microscope (Olympus AX70TRF; Olympus, Tokyo, Japan). Nuclei were stained with bisbenzimide (1 µg/ml).

### Electron microscopy

In prior work [5] we generated 600 electron micrographs from three control and three Dicer KO bladders (10 weeks after tamoxifen). Here, using ImageJ (NIH, Bethesda, MD, USA), we analyzed collagen fibril diameters and thickness of the basal membrane surrounding smooth muscle cells in this collection of micrographs. Thickness of the basal lamina was measured on muscle cells where the contractile filaments appeared as perfectly rounded structures to ensure that muscle cells were cut orthogonally to their longitudinal axis. Only collagen fibril profiles that appeared perfectly circular were measured. 

### Passive mechanical properties

After removal of the mucosa, equatorial strips of control and Dicer KO mouse detrusors were prepared [28] and mounted between stainless steel pins in four-channel myograph chambers (610 M; Danish MyoTechnology, Aarhus, Denmark) filled with aerated HEPES-buffered Krebs solution (sodium chloride, 135.5 mM; potassium chloride, 5.9 mM; magnesium chloride, 1.2 mM; glucose 11.6 mM; HEPES, 11.6 mM; pH 7.4 at 37°C). Ca^2+^-free conditions were used to avoid contribution from active contractile processes and length was increased in a stepwise fashion. Force was determined after 10 min of equilibration at each length. Passive force per cross-sectional area (stress) was calculated from length and weight [5,28] and circumferences were calculated using the percentage that the strip constituted of the complete circumference during dissection. Stresses at specified circumferences were obtained from the best fit of a cubic binomial function. For treatment with elastase, rat detrusor strip were prepared and incubated for 30 min with 10 µg/ml elastase (Sigma-Aldrich). They were then mounted in the myograph and subjected to the same experimental protocol as the mouse strips. 

### Statistical analysis

Significance analysis of microarrays was done using TMEV v.4.0 software and returned a q value [30]. A q value of 0 was considered significant. TFBS analysis was done as described [29]. All other statistical comparisons were done using GraphPad Prism version 5.02 for Windows (GraphPad Software, San Diego California USA). Single comparisons were made using Student’s t test. Multiple comparisons were made with analysis of variance followed by Dunnett's Multiple Comparison Test or Tukey's Multiple Comparison Test. In situations with unequal variances, the Welch correction was used. Correlations were tested using linear regression analysis. A *p* value <0.05 was considered significant. 

## Results

### Outlet obstruction activates TGF-β signaling

**Figure 1:**
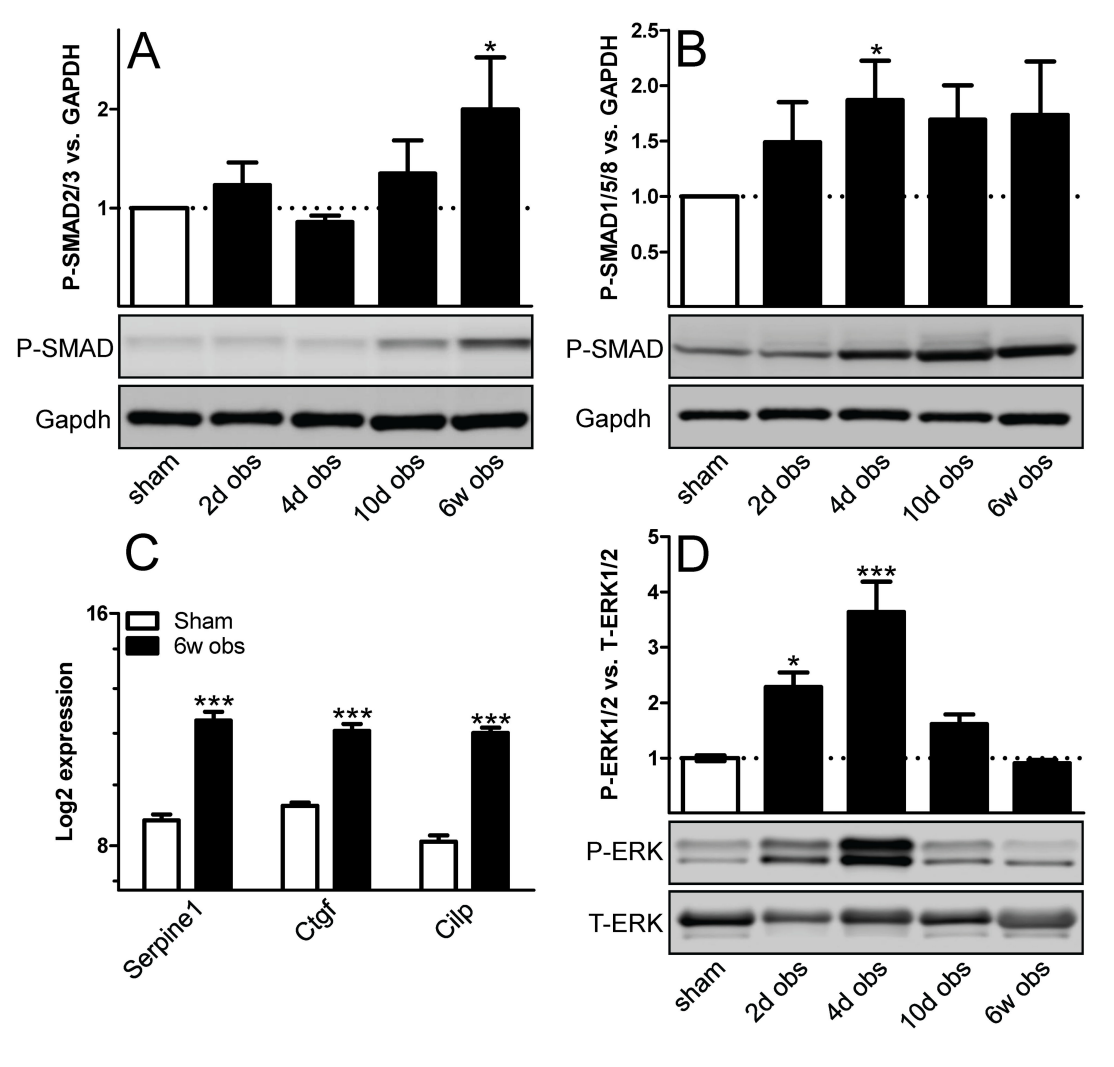
Rat bladder outlet obstruction leads to SMAD and extracellular-signal-regulated kinase (ERK) phosphorylation and to increased transcript levels of established transforming growth factor-β (TGF-β) targets. Phosphorylation of SMAD proteins (A) SMAD2/3 and (B) SMAD1/5/8 was measured by western blotting using phosphorylation-sensitive antibodies at various times following outlet obstruction. Gapdh was used as loading and normalization control. Protein expression was normalized first to Gapdh and then to the mean for the sham-operated controls. (C) Transcript levels for three established TGF-β targets are shown at 6 weeks of obstruction. The fold changes were large so the log_2_ expression is shown on the y-axis. Data is from the mRNA microarray described in further detail in the text. (D) Time-course of ERK1/2 activation. All samples: n=6-8. * *p* < 0.05 versus sham. *** *p* < 0.001 versus sham.

To investigate if TGF-β signaling is activated following outlet obstruction, rat bladders were harvested at various time points following surgical obstruction of the urethra, and phosphorylated SMAD proteins (P-SMAD) was measured. P-SMAD2/3 started to increase compared to sham at 10 days and was significantly elevated at 6 weeks (Figure 1A). In contrast, P-SMAD1/5/8 was increased already at 4 days and did not change further thereafter (Figure 1B). Transcript levels for established TGF-β targets were increased at 6 weeks (Figure 1C). We also assayed extracellular-signal-regulated kinase phosphorylation (P-ERK1/2), which may bias TGF-β signaling towards the SMAD1/5/8 pathway, and found it to be increased at 2 days and 4 days, respectively (Figure 1D).

### Outlet obstruction and TGF-β reduce miR-29 expression

**Figure 2:**
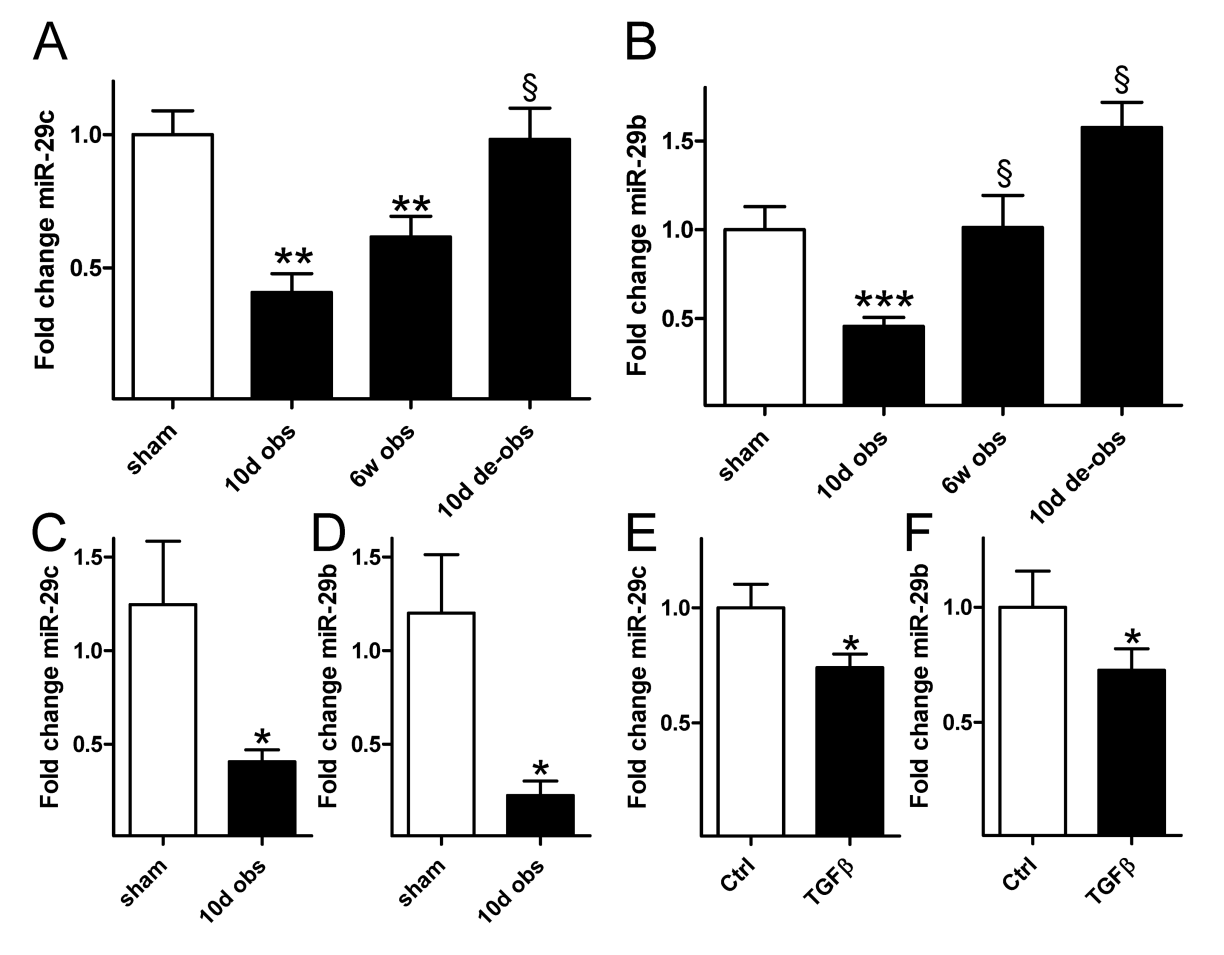
Outlet obstruction and transforming growth factor β (TGF-β1) stimulation leads to reduced expression of miR-29. Time courses of (A) miR-29c and (B) miR-29b expression following rat bladder outlet obstruction. Data are from miRNA microarrays (*n*=6−8). Independent confirmation of reduced (C) miR-29c and (D) miR-29b in the obstructed bladder by real-time quantitative polymerase chain reaction (*n*=6). Expression of (E) miR-29c and (F) miR-29b in vehicle-treated (control) and TGF-β1-treated human urinary bladder smooth muscle cells. Cells were treated with 5 ng/ml TGF-β1 in passages 3−5 (six independent replicates using cells from one individual). * *p* < 0.05 versus control in C through F. ** *p* < 0.01 versus sham in A. *** *p* < 0.001 versus sham in B. § *p* < 0.05 versus 10 d of obstruction in A and B. De-obs = de-obstructed; sham = sham-operated controls.

We next set out to determine miR-29 expression. Because a comprehensive profile of differentially expressed miRNAs in outlet obstruction would be valuable for future studies we used miRNA arrays comparing sham-operated rat bladders with those obstructed for 10 days and 6 weeks. One group of rats was also obstructed for 6 weeks and then reoperated to remove the obstruction. This group was included to assess reversibility. MiR-29b and miR-29c were among the 63 differentially expressed miRNAs in outlet obstruction (q=0; n=6−8; GEO accession number GSE47080). MiR-29a did not change significantly (0.9-fold vs. sham, q=19). Time courses of expression for miR-29c and miR-29b from the microarray experiment are depicted in Figure 2A and 2B. Real-time quantitative PCR for miR-29c and miR-29b (Figure 2C and D) confirmed reduced expression of both miRNAs at 10 days.

We next tested whether TGF-β1 reduces miR-29 expression using cultured smooth muscle cells from human bladder. Stimulation with TGF-β1 for 48h led to reduced expression of miR-29c and miR-29b (Figure 2E and F).

### MiR-29 target mRNAs change in outlet obstruction

**Figure 3:**
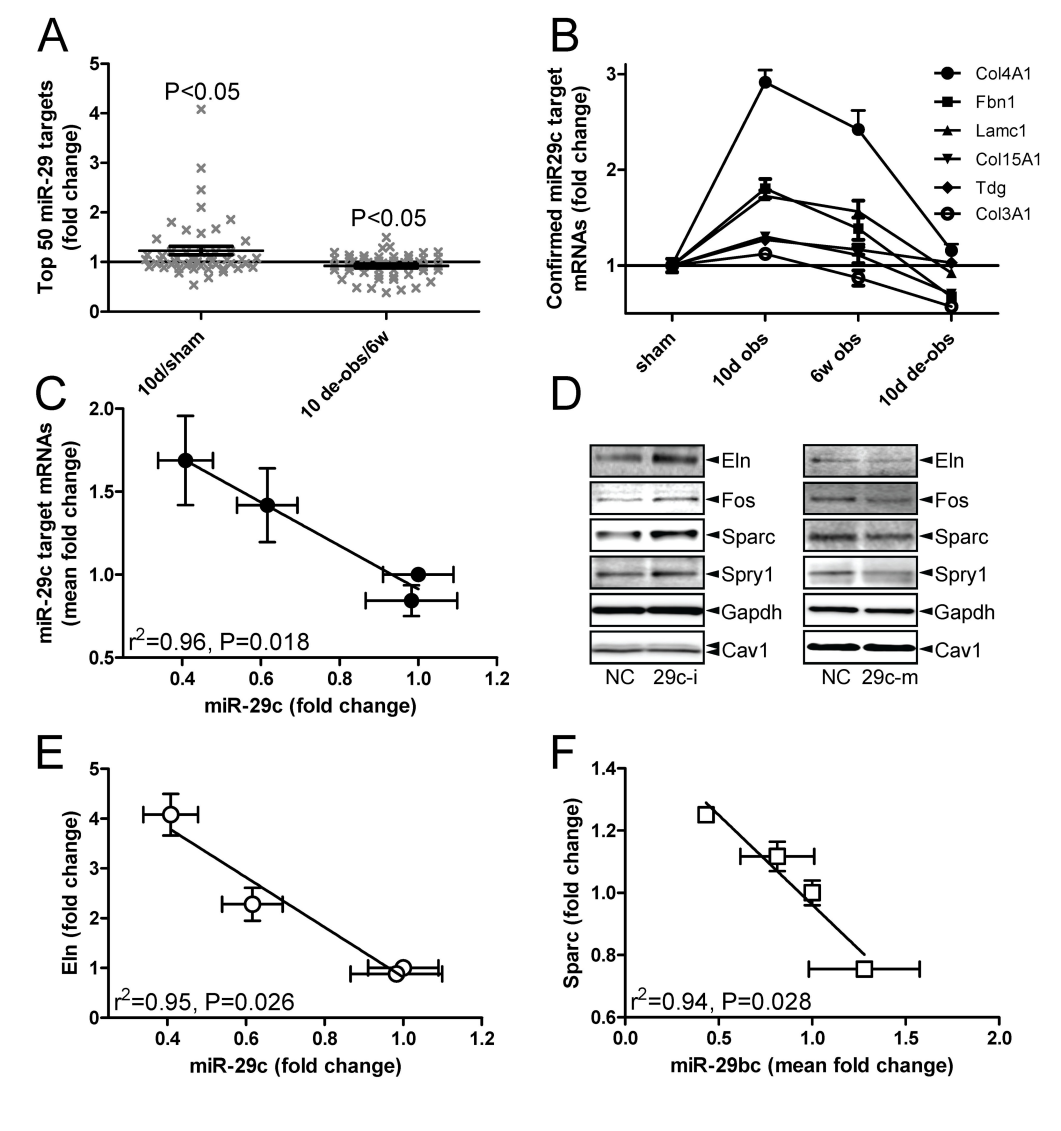
Repression of miR-29 during outlet obstruction is associated with increased levels of miR-29 target messenger RNAs (mRNAs). Gene expression was assessed (*n*=6−8) at 10 days and at 6 weeks following outlet obstruction using microarrays and compared with sham-operated controls and with rats where the obstruction had been removed for 10 days. (A) Relative increase (left vs. sham) and decrease (right vs. 6 weeks) of the top 50 mRNA targets of miR-29 when miR-29 was repressed at 10 days and when miR-29 recovered after de-obstruction (c.f. Figure 2A and B). The statistical comparison was made versus a theoretical value of 1 (no change) using Student’s t test. (B) Time courses of mRNA expression for six confirmed miR-29c targets (official symbols are given in the legend). Fold changes are versus sham. (C) Correlation between miR-29c and the mean fold change of the target mRNAs depicted in Figure 3B. (D) Western blots for miR-29 targets in human urinary bladder smooth muscle cells transfected with negative control and miR-29c-inhibitor (left row). Gapdh and caveolin-1 were used as loading controls. Cells were used in passages 3−5 and harvested 96 h after transfection. Targets shown were significantly changed (one-tailed Student’s t test) in independent replicates (*n*=3−5) using cells from one individual. Right row in D shows effect of miR-29c mimic. (E) Inverse correlation between fold change of miR-29c (vs. sham) and fold-change of Eln (vs. sham) in outlet obstruction/de-obstruction. (F) Inverse correlation between the mean fold change of miR-29b and miR-29c (both vs. sham) and fold-change of Sparc (vs. sham). In panels C, E and F the sham-operated controls are represented by the symbols at x=1, y=1 whereas 10 d obstructed bladders are represented by the leftmost symbols. The remaining two symbols represent 6 weeks obstructed and de-obstructed bladders, respectively.

To address whether reduced miR-29b/c following outlet obstruction was associated with altered expression of target mRNAs we did an mRNA microarray experiment. Bladders obstructed for 10 days and 6 weeks as well as de-obstructed bladders and sham-operated controls were again included (six to eight in each group; GEO accession number GSE47080). More than 1000 transcripts were differentially expressed at 10 days. We mined the mRNA arrays for the top 50 predicted targets (www.targetscan.org) of all differentially expressed miRNAs. MiR-1 (not shown), miR-29b, and miR-29c returned significant associations with target mRNA levels. The initial association found for miR-29b/c is illustrated in Figure 3A which shows that miR-29b/c targets were elevated at 10 days (vs. sham), a time point when miR-29 was reduced (c.f. Figure 2A and B). When miR-29b/c increased on de-obstruction (vs. 6 weeks) the same mRNAs declined (compare Figure 2A, B with Figure 3A). Genes encoding matrix proteins, such as collagen type IV, α1 (Col4a1), fibrillin 1 (Fbn1), laminin γ-1 (Lamc1), and tropoelastin (Eln), were represented among the top 50 targets. 

A previous microarray study [31] with close to genome-wide coverage showed that overexpression of miR-29c reduced six mRNAs: Col4a1; Fbn1; Lamc1; collagen, type XV, α1 (Col15A1); thymine-DNA glycosylase (Tdg); and collagen, type III, α1 (Col3A1). Expression of all six of these targets peaked at the nadir of miR-29c expression (compare Figure 3B with Figure 2A), and they declined sharply on de-obstruction when the miR-29c level recovered. We plotted the mean expression of these mRNAs versus the mean miR-29c level in sham-operated control bladders, after 10 days and 6 weeks of obstruction, and after 10 days of de-obstruction. A significant inverse correlation was found (Figure 3C). 

### Transfection of miR-29c inhibitor and mimic

MiRNA inhibitors are small RNA molecules designed to inhibit microRNAs. Human detrusor cells were transfected with miR-29c inhibitor and eight validated miR-29 targets, most of which were represented among the top 50 predicted targets in Figure 3A, were examined using western blotting. Collagens are large insoluble complexes that cannot be assessed by western blotting and were therefore avoided. Mature elastin fibrils can similarly not be measured by western blotting so we examined the precursor protein tropoelastin. Four of the eight selected miR-29 targets, including Eln (elastin or tropoelastin), Fos (also known as c-Fos), Sparc (osteonectin) and sprouty homolog 1 (Spry1), were significantly increased following inhibitor transfection (Figure 3D, left row). With the possible exception for tropoelastin, transfection of miR-29c mimic was associated with a reduction of these proteins (Figure 3D, right row). In view of this outcome we tested if Eln and Sparc mRNAs were individually correlated with miR-29 in outlet obstruction. Eln correlated significantly with miR-29c (Figure 3E) and with the mean of miR-29b and miR-29c (not shown). Sparc correlated with the mean of miR-29b and miR-29c (Figure 3F). We also predicted the free energies of miR-29c binding to proximal sites in the Eln and Sparc 3’UTRs and found them to be within the range of authentic miRNA-target pairs (Figure S1A, B). 

### Outlet obstruction activates c-Myc and NF-κB

**Figure 4:**
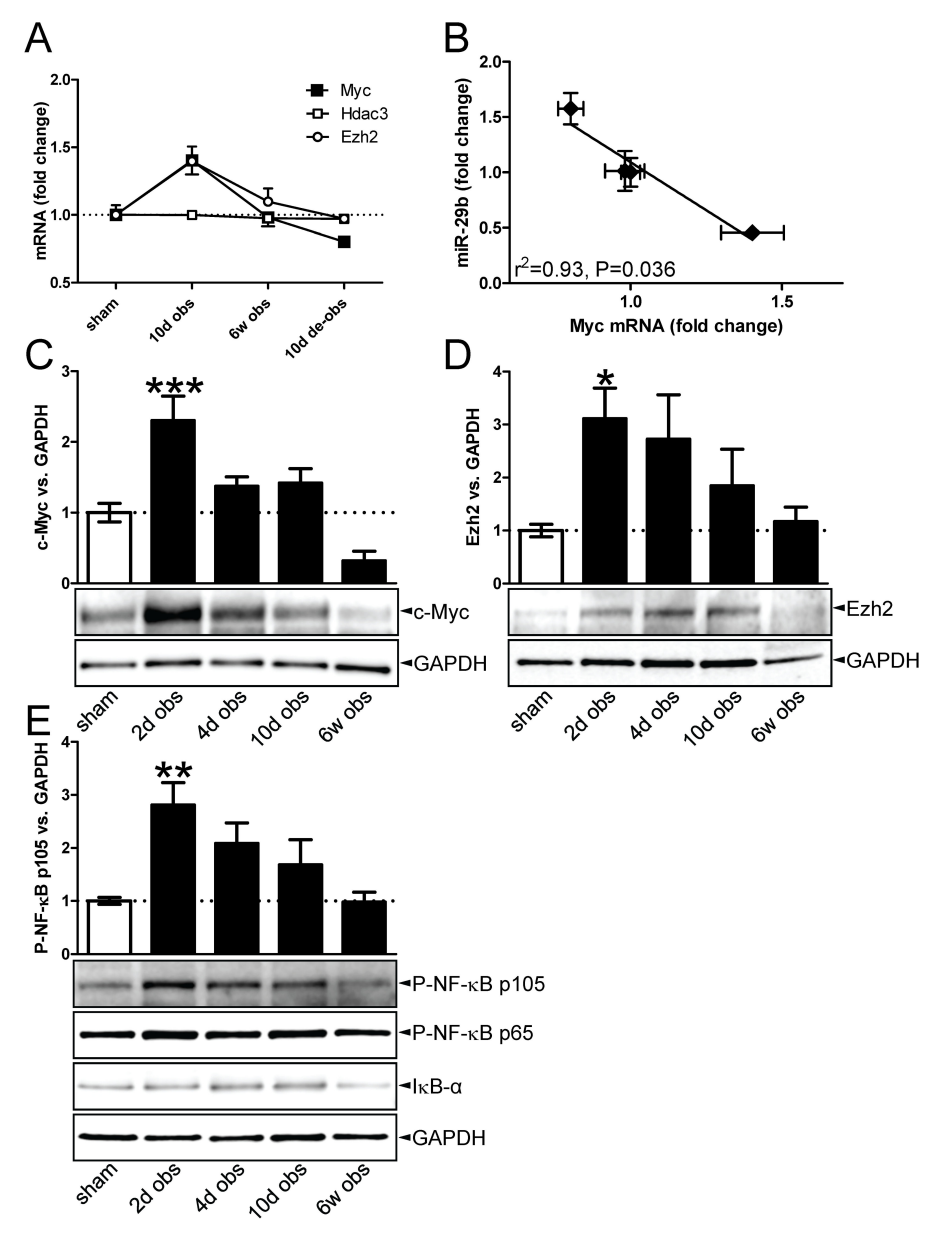
c-Myc accumulation in outlet obstruction. (A) Fold change (vs. sham) of c-Myc (Myc), Hdac3 and Ezh2 mRNAs in outlet obstruction and de-obstruction (n=6-8). (B) Inverse correlation between miR-29b and Myc mRNA level (both expressed relative to sham) in outlet obstruction and its reversal (n=6-8). The leftmost symbol represents 10 d obstruction, the rightmost symbol represents de-obstruction, and the symbols close to x=1 represent sham and 6 weeks of obstruction, respectively. Western blots and bar graph summaries showing increased c-Myc (C) and Ezh2 (D) levels in outlet obstruction (n=6 for both). (E) Western blots and bar graph summary showing increased phospho-NF-κB p105 in outlet obstruction (n=6). Blots for phospho-NF-κB p65 and IκB-α are shown without bar graph summaries as no significant differences were seen.

SMAD3 activation, which is known to be involved in TGF-β-mediated repression of miR-29, was not significantly increased at 10 days when miR-29b and miR-29c appeared to be maximally repressed. MiR-29 is also known to be repressed by c-Myc and NF-κB. Using the available mRNA microarray data we therefore did a transcription factor binding site analysis (TFBS) at 10 days of obstruction searching for indirect evidence of c-Myc and NF-κB activation. Significant enrichment of c-Myc binding sites (*p*=0.03), of binding sites for c-Myc and its partner Max (*p*=0.01) and of NF-κB binding sites (*p*=10^-4^) were found in the promoters of our 10 day dataset. SMAD3 binding sites were not significantly enriched (*p*=0.22). This supported the possibility that c-Myc and NF-κB, but not SMAD3, might be involved in the repression of miR-29b and miR-29c at 10 days.

c-Myc-mediated repression of miR-29 involves a complex consisting of c-Myc (Myc), histone deacetylase 3 (Hdac3) and enhancer of zeste homolog 2 (Ezh2) which binds to conserved sequences in the promoters of the miR-29a/b1 and miR-29b2/c genes [18]. To substantiate the involvement Myc/Ezh2 we plotted their mRNA levels during obstruction/de-obstruction (Figure 4A). This demonstrated an increase of Myc and Ezh2 mRNAs at 10 days (c-Myc 10 days vs. sham: q=0.06, *p*=0.006; Ezh2 10 days vs. sham: q=0.61, *p*=0.016). The Myc mRNA declined below the control level on de-obstruction, resulting in a significant and inverse correlation with miR-29b (Figure 4B). Hdac3 on the other hand remained unchanged. We therefore examined c-Myc and Ezh2 proteins using western blotting. Quantification showed that the ~65 kDa c-Myc band increased shortly after obstruction. It was subsequently maintained at a level slightly higher than that of the sham-operated controls and thereafter it dropped (Figure 4C). Ezh2 was elevated at 2 days (Figure 4D) and then declined progressively to control level. We also blotted for phospho-NF-κB p105 and found it to be increased within the first few days of obstruction (Figure 4E). Phospho-NF-κB p65 and IκB-α did not change significantly compared to sham at any time point (Figure 4E and data not shown). 

### Bladder outlet obstruction increases miR-29 target protein levels

**Figure 5:**
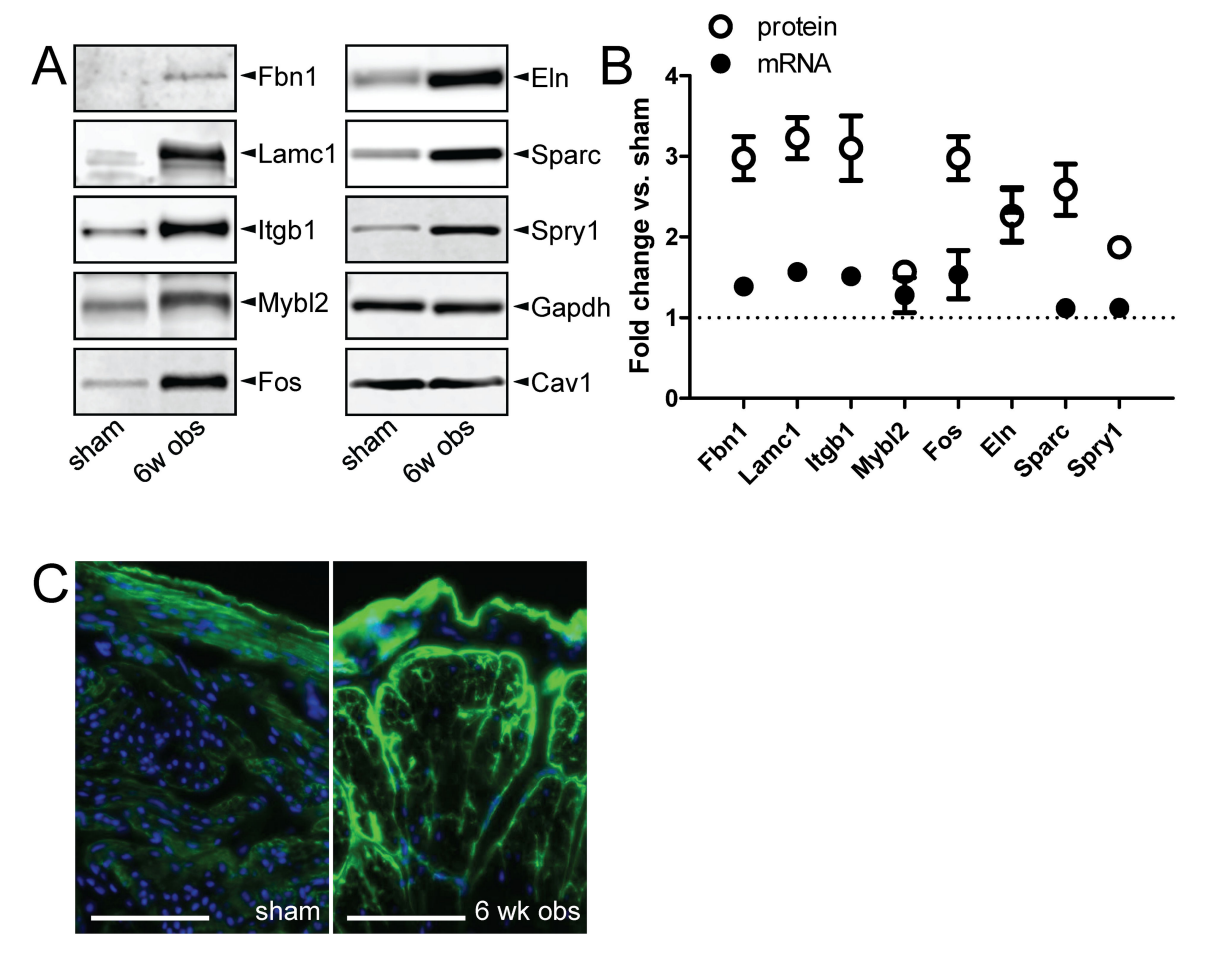
Repression of miR-29 after outlet obstruction is associated with increased levels of miR-29 target proteins. (A) Western blots for eight miR-29 targets in sham-operated control bladders and at 6 weeks of obstruction. Gapdh and Cav1 were used as loading controls (*n*=6 throughout). (B) Relative fold change at 6 weeks of obstruction (vs. sham) of miR-29 target messenger RNAs (mRNA; black circles) and proteins (white circles). The fold change of mRNA compared to the fold change of the protein was significantly different throughout, except for Eln and Mybl2. MRNA expression is from the microarrays (*n*=6−8), and protein expression is from the western blots in A. Protein expression was normalized first to Gapdh and then to the mean for the sham-operated controls. (C) Immunofluorescence staining for collagen IV (col4a1, green) in sham-operated control and 6-wk obstructed detrusor. Scale bars represent 100 µm. The fluorescent pear-shaped profile in the center of the 6 weeks specimen represents the outline of a muscle bundle. Individual cell profiles are visible within the bundles. Nuclei are stained blue and the outer bladder surface is facing upward in both micrographs.

The impact of miR-29 in outlet obstruction is likely underestimated by measuring levels of mRNAs because an important mechanism of miRNAs is translational repression. Thus we measured the eight validated miR-29 target proteins (the same ones measured after inhibitor transfection) at 6 weeks of obstruction. We blotted for Fbn1, Lamc1, intergrin β1 (Itgb1), V-myb myeloblastosis viral oncogene homolog (avian)-like 2 (Mybl2; also known as B-Myb), Fos, Eln, Sparc and Spry1, and all were elevated compared to Gapdh and caveolin-1 (Cav1) which were used as loading controls (Figure 5A). In all cases except for Eln and Mybl2, protein expression was increased more than mRNA expression; for example, Itgb1 was increased 2.8-fold (±0.3) at the protein level but only 1.5-fold (±0.05) at the mRNA level (Figure 5B). We next labeled collagen IV (Col4a1) on detrusor sections using indirect fluorescence. Much brighter staining was revealed between smooth muscle cells and around muscle bundles in obstructed compared to control bladders (Figure 5C).

### Depletion of miRNAs reduces detrusor compliance independent of outlet obstruction

**Figure 6:**
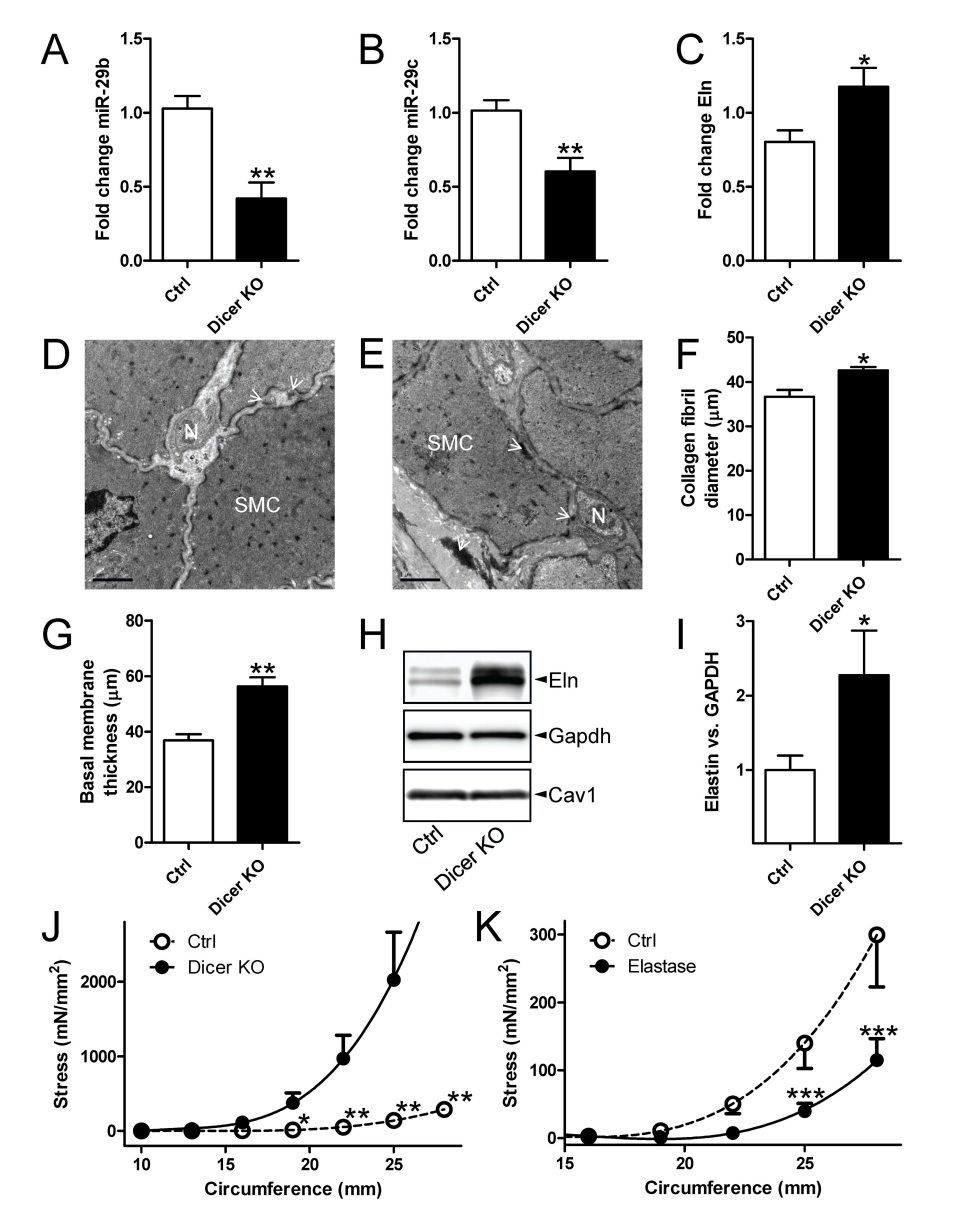
MiR-29 depletion by genetic deletion of Dicer in smooth muscle increases detrusor stiffness in Ca^2+^-free conditions. Real-time quantitative polymerase chain reaction (*n*=5-7) for miR-29b (A), miR-29c (B) and elastin (Eln, C) in control (Ctrl) and Dicer knockout (KO) mouse bladders. Electron micrographs from control (D) and Dicer KO (E) detrusor. SMC: smooth muscle cell; N: neural varicosity. White arrowheads point to elastin fibrils. Scale bars represent 1 µm. Quantitative morphometry of collagen fibril diameters (F) and basal membrane thickness (G). (H) Western blots for Eln, Gapdh and Cav1 in detrusors from control and smooth muscle-specific Dicer knockout (KO) mice. Summarized data on Eln expression in control and Dicer KO bladders (I, *n*=12). (J) Passive circumference-stress curves in Dicer KO and control bladder strips (*n*=12). (K) Passive circumference-stress relations in control and elastase-treated rat detrusor strips (n=4).

We next examined whether manipulation of miRNA expression *in vivo* can affect matrix composition and detrusor compliance independent of outlet obstruction. For this we used mice with smooth muscle-specific inactivation of Dicer (Dicer knockout [KO]). We first examined expression of miR-29b and miR-29c following 5 weeks of Dicer deletion and found both miRNAs to be reduced (Figure 6A, B). The partial reduction likely reflects the heterogeneous cell composition of the bladder, but time may also be a factor. The Eln mRNA level was also increased at this time (Figure 6C). We next searched for evidence of matrix remodeling at 10 weeks following Dicer deletion using electron microscopy (Figure 6D, E). Collagen fibril diameters were modestly increased (Figure 6F) as was the thickness of the basal lamina surrounding the smooth muscle cells (Figure 6G). Elastin fibrils which can be seen extracellularly as large electron dense aggregates with small fibers in their periphery, appeared more prominent in Dicer KO bladders (compare Figure 6D and E, white arrowheads), but were too rare to allow for reliable morphometric quantification. Tropoelastin, when examined using western blotting, was however increased in comparison with control mouse detrusor (Figure 6H, summarized data in I). In keeping with the observed matrix remodeling, mechanical experiments showed increased passive force per cross-sectional area in Dicer KO compared to control detrusor strips at all circumferences exceeding 20 mm (Figure 6J). We finally tested if elastin degradation using elastase had the opposite effect. Indeed, treatment of rat detrusor strips with elastase *in vitro* reduced passive force per cross-sectional area at circumferences exceeding 25 mm (Figure 6K).

## Discussion

Impressive progress in miRNA-targeted chemistries has been made in recent years [3]. This supports the advent of a novel therapeutic modality. The biology of specific miRNAs is far from completely understood, and their role in lower urinary tract pathologies other than cancer is only beginning to be appreciated [5,6,32,33]. The current study provides support for a role of miRNAs in gene expression and compliance regulation in the urinary bladder following outlet obstruction. We propose that bladder distension leads to repression of miR-29 via three distinct mechanisms and that this has an impact on tropoelastin and Sparc synthesis and on tissue mechanical properties. This in turn may allow the tissue to cope with the mechanical distension caused by accumulation of residual urine (Figure S2). This model can perhaps be generalized, as suggested by findings in the infarcted heart [10] and in aortic aneurysm progression [8], both of which are clinical conditions with considerable organ distension.

Our starting hypothesis was that TGF-β/SMAD3 signaling would repress miR-29 in outlet obstruction. This hypothesis was based on a handful of prior studies demonstrating increased mRNA levels for different TGF-β isoforms shortly after outlet obstruction (e.g. 24), and on the documented repression of miR-29 by TGF-β/SMAD3 [11]. In accordance with those prior studies, our mRNA microarrays revealed increased levels for both TGF-β2 and TGF-β3 at 10 days of obstruction (2.6- and 3.3-fold versus sham, q=0 for both, GEO GSE47080). We found however, that SMAD2/3 phosphorylation increased only after prolonged obstruction (6 weeks). The ability of TGF-β to activate noncanonical SMAD signaling in a Ras/MEK/ERK-dependent manner is well established [34-36], and in keeping with this possibility, we also observed ERK1/2 phosphorylation coinciding with early SMAD1/5/8 phosphorylation. One possibility, therefore, is that increased TGF-β mRNA levels at 10 days reflect production of bioactive TGF-β that signals via the noncanonical (SMAD1/5/8) pathway. Another possibility is that the newly synthesized TGF-β is sequestered in inactive form in the matrix by latent TGF-β-binding proteins (LTBP1-4), and that the early SMAD1/5/8 activation is a consequence of BMP-signaling. This latter alternative is supported by the fact that LTBP2 mRNA increased more than any of the TGF-β isoforms at 10 days (c.f. GEO GSE47080). Our findings do not provide any further guidance as to which possibility is correct, but they do indicate that SMAD3, which is specific for the miR-29b2/c gene [11], may be involved in chronic repression of miR-29c, but not in its acute reduction. 

Our lack of evidence for SMAD2/3 activation at 10 days, when miR-29b and miR-29c appeared maximally repressed, forced us to consider alternative mechanisms. c-Myc and NF-κB are also known to repress miR-29 [11,18], and both pathways have previously been shown to be activated in outlet obstruction and by mechanical distension [37-39]. c-Myc, Hdac3 and Ezh2 form a repressive complex that binds to conserved sequences in the miR-29a/b1 and miR-29b2/c promoters [18]. Our TFBS analysis indicated enrichment of c-Myc sites in the promoters of differentially regulated genes at 10 days. A correlation between the c-Myc mRNA and miR-29b was moreover seen, and we directly demonstrate accumulation of c-Myc and Ezh2 using western blotting. Taken together these findings strongly support the view that the aforementioned repressor complex, assembled upon accumulation of c-Myc/Ezh2 in outlet obstruction, regulates miR-29b in the detrusor. 

NF-κB stimuli can cause nuclear accumulation of NF-κB by IκB kinase-mediated phosphorylation of p105 [40]. This mechanism appeared to be operative in the rat urinary bladder since a transient peak of phospho-NF-κB p105 was detected. IκB-α, which when degraded liberates transactivation-competent dimers, on the other hand appeared to be stable throughout our time-course. We also found enrichment of NF-κB sites in our TFBS analysis. Together, these findings agree well with the activation of NF-κB seen previously in outlet obstruction using probes encompassing the consensus binding sequence of NF-κB [38,39]. We therefore propose that c-Myc/Hdac3/Ezh2 and NF-κB are jointly responsible for repression of miR-29b and miR-29c at 10 days and that SMAD3 is responsible for the sustained repression of miR-29c. For miR-29c this would give three waves of repressive influences that together ensure a rapid yet longstanding decrease in outlet obstruction. 

The experimental support for an impact of miR-29 on protein synthesis in the bladder following outlet obstruction extends well beyond a significant correlation between miR-29b/c and target mRNAs. For tropoelastin, whose mRNA correlated inversely and significantly with miR-29 in outlet obstruction, we found that transfection of a miR-29c inhibitor resulted in increased tropoelastin expression. We moreover demonstrate that genetic deletion of smooth muscle miRNAs increases tropoelastin in the mouse bladder *in vivo*. Thus, several independent lines of evidence support a regulatory role of miR-29 in tropoelastin synthesis in the bladder, consistent with the large number of miR-29 binding sites in its mRNA [10,12]. Sparc, Spry and Fos were similarly reciprocally regulated by miR-29c inhibitor and mimic, and increased in outlet obstruction. 

Some predicted and confirmed miR-29 targets, including Fbn1, which is an integral part of the elastic fiber meshwork, and Lamc1, a protein present in the basement membrane, were increased in outlet obstruction but were not significantly affected at the protein level following inhibitor transfection (not shown). There are many possible explanations for this discrepancy. For instance, cell culture *per se* may promote a proliferative phenotype with activation of c-Myc. This would repress miR-29, and hence it would be more difficult to see an effect of miR-29 inhibition. Considerably more time was moreover allowed for de-repression of miR-29 targets in the *in vivo* setting (6 wk vs. 96 h). An emerging theme, finally, in miRNA biology is that the impact of miRNAs is modest in homeostatic conditions, becoming pronounced only in conditions of stress [3], such as in outlet obstruction, when mRNA and protein turnover rates are drastically increased. Together, these circumstances may well explain the more widespread apparent impact of miR-29 repression in outlet obstruction (8/8 examined target proteins increased) compared to inhibitor transfection (4/8 examined target proteins increased).

Detrusors from smooth muscle-specific Dicer knockout mice were used to examine if reduction of miR-29 *in vivo* increases tropoelastin expression. We found that Dicer knockout reduced miR-29b and miR-29c by about half and increased tropoelastin expression. It may be argued that a miRNA other than miR-29 is responsible for altered tropoelastin expression in Dicer KO bladders. Of the 30 miRNAs that are highly expressed in the mouse detrusor [5,41] none except miR-29 is predicted to target tropoelastin. Unlike other miRNAs, miR-29 also targets a large battery of collagens, including collagens I and III [10]. We have not done an exhaustive characterization of the matrix composition in Dicer knockout bladders, but it is clear from our prior morphometric analyses that the extracellular matrix is increased, as indicated by a significant increase in the distance between smooth muscle cells [5]. Here we also demonstrate an increased thickness of the Col4a1-containing basal lamina surrounding smooth muscle cells and a modest increase of the collagen fibril size. Together, these findings support the view that repression of miR-29, independent of surgical obstruction of the urethra, leads to matrix remodeling.

We hypothesized that miR-29 repression may contribute to increased detrusor stiffness in outlet obstruction. Several prior studies have demonstrated that outlet obstruction increases bladder stiffness (e.g. 19). We therefore examined if miRNA repression affects the passive mechanical properties of the detrusor in the absence of outlet obstruction using Dicer KO bladders. For a bladder strip in tension, the axial stiffness (in N/m) is defined by the product of its cross-sectional area and its elastic modulus (Young's modulus) divided by its length. The elastic modulus reflects properties intrinsic to the tissue and changes if the composition of the tissue changes. We found passive force per cross-sectional area to increase more steeply in Dicer KO compared to control detrusor strips. It follows that the elastic modulus is increased. Because the weight and the relative thickness of the detrusor layer of Dicer KO bladders are unchanged [5] we can conclude that the passive stiffness of the detrusor increases upon knockdown of smooth muscle miRNAs. The elastic modulus will, at least theoretically, increase if some stiff material (with a high elastic modulus) increases relative to a more compliant material (with a low elastic modulus). That is, when fibrillar matrix components increase relative to a more compliant material, such as soluble protein (e.g. GAPDH, or indeed most of the proteins encoded by the reference genes on a microarray), then the elastic modulus and the stiffness should increase. In support of this reasoning it has been demonstrated that elastase, which degrades elastin, reduces both stiffness and the elastic modulus of smooth muscle preparations [42,43]. Here we directly demonstrate that this also applies to bladder smooth muscle. Increased elastin content may therefore contribute to altered passive mechanical properties in Dicer KO detrusors, but collagens, or indeed any other target of miRNAs, may also play a role. Even if the critical matrix change responsible for the altered stiffness in Dicer KO detrusors has not been definitively identified, it is clear that miRNA repression increases stiffness. This in turn counteracts distension (Figure S2, circle six). 

The earliest studies documenting matrix remodeling in the urinary bladder after outlet obstruction showed increased elastin content [44]. Subsequent work demonstrated a three-fold increase in total collagen, but because of massive detrusor hypertrophy/hyperplasia the concentration of collagen actually falls by about half [45]. Later studies have indicated increases of type I and type III collagens [46] (reviewed by [23]). We did not focus our efforts on these collagens because total levels of fibrillar collagens are not easily quantified using western blotting. Type I and type III collagens are however established targets of miR-29 [10], and their mRNAs were largely unchanged at 10 days and at 6 weeks (Col1a1 at 10 days: up by 15%, q=2.7, p=0.05; Col3a1 at 10 days: up by 12%, q=8.7, p=0.12). Therefore, reduction of miR-29b and miR-29c could well promote collagen production at an unchanged mRNA level at these times. The matricellular protein Sparc is a confirmed miR-29 target [13] that influences collagen fibril morphology and function [47]. De-repression of Sparc may thus also contribute to a miR-29-mediated change of detrusor stiffness in outlet obstruction.

Col4a1 is, similar to collagens I and III, difficult to assess using standard blotting techniques because non reducing conditions are required and because the precursor protein migrates at a very high molecular weight. However, because no prior study had demonstrated increased deposition of this basal membrane collagen in outlet obstruction, we chose to confirm the change in Col4a1 with immunofluorescence staining. This revealed a pronounced increase around individual cells and around muscle bundles in obstructed bladders, consistent with its increased mRNA level and with the fact that it is a miR-29 target [48]. We did not examine Col4a1 in the transfection experiment for the reasons outlined above, but, using electron microscopy, we found that the basal membrane surrounding smooth muscle in Dicer KO bladders cells had an increased thickness. Added to the correlation between miR-29c and Col4a1 mRNA this favors a causal relationship between the repression of miR-29 and the increase of Col4a1 in outlet obstruction. 

Several of the miR-29 targets that we studied, including Col15a1, Tdg and Spry1, have not been considered previously in the context of hypertrophic growth and remodeling of the bladder. Currently, we can only speculate on the role of these proteins in outlet obstruction, but it is of considerable interest that the miR-29 target Spry1 [14], which is an established ERK1/2 inhibitor [49], increases after prolonged outlet obstruction. This may allow for feedback inhibition of ERK1/2 activity. In keeping with this possibility, ERK1/2 activity increased 4-fold at 4 days and then returned to control level at 6 weeks despite maintained distension. Further work is required to elucidate whether this phasic behavior may be explained by Spry1 accumulation. Mybl2 (B-Myb) is, similar to ERK1/2, considered to play a role in cell cycle regulation [50], and a role in bladder growth may therefore tentatively be assigned to this protein.

##  Conclusions

Taken together, the present study shows that bladder outlet obstruction, such as that seen in elderly men with enlarged prostate glands, leads to reduced expression of miR-29b and miR-29c in the bladder and that this is associated with increased expression of miR-29 targets, including the matrix proteins elastin and Sparc. We also demonstrate that genetic depletion of miRNAs, including miR-29, increases bladder elastin expression and stiffness independently of outlet obstruction and that miR-29 inhibitor transfection *in vitro* replicates several of the expression changes associated with miR-29 repression in outlet obstruction. Combined, these findings provide support for our hypothesis that miR-29 reduction contributes to increased protein synthesis in the bladder following outlet obstruction and that this in turn influences matrix properties and stiffness (Figure S2). The molecular pathways that we find are activated by bladder distension may be distension-responsive in other smooth muscle-containing tissues, including blood vessels.

## Supporting Information

Figure S1 **Free energies (ΔG^0^) of miR-29c binding to the proximal 3’UTR site in rat, mouse and human elastin (Eln, panel A) and osteonectin (Sparc, panel B), respectively.** Base-pairing in the seed region is indicated by vertical lines. Additional nucleotides are also responsible for the binding strength. The free energies were obtained using the FindTar3 database. The binding sites shown, and the more distal sites, are all well conserved between species.
(TIFF)

Figure S2 **Flow chart showing the model proposed for miR-29 repression in outlet obstruction and for miR-29-mediated matrix remodeling and altered passive mechanical properties.** Outlet obstruction such as seen in elderly men with enlarged prostate glands and in children with urethral valves leads to (1) distension of the detrusor. This in turn (2) activates multiple signaling pathways including c-Myc, NF-κB and TGF-β/SMAD3 that in turn repress miR-29. The reduced level of miR-29 leads to increased levels of mRNAs encoding extracellular matrix proteins (3), including elastin and Sparc (osteonectin), but possibly also collagens and fibrillin-1. The resulting protein synthesis and matrix deposition (4) leads to increased detrusor stiffness (5) (and increased elastic modulus) which counteracts (6) further distension. The proposed model fits the data presented in this article, but alternative interpretations are possible and steps upstream of miR-29 repression need *in* *vivo* corroboration. The electron micrographs were captured at a magnification of x6000 and are from control (bottom) and Dicer KO (top) detrusors. The extracellular matrix between muscle cells has been highlighted using transparent red color and scale bars represent 5 µm.
(TIFF)
