# Supplementary figures and images for: Mir-29 Repression in Bladder Outlet Obstruction Contributes to Matrix Remodeling and Altered Stiffness
Source: PLoS One. 2013 Dec 10;8(12):e82308. doi: 10.1371/journal.pone.0082308 (PMC3858279; doi:10.1371/journal.pone.0082308)

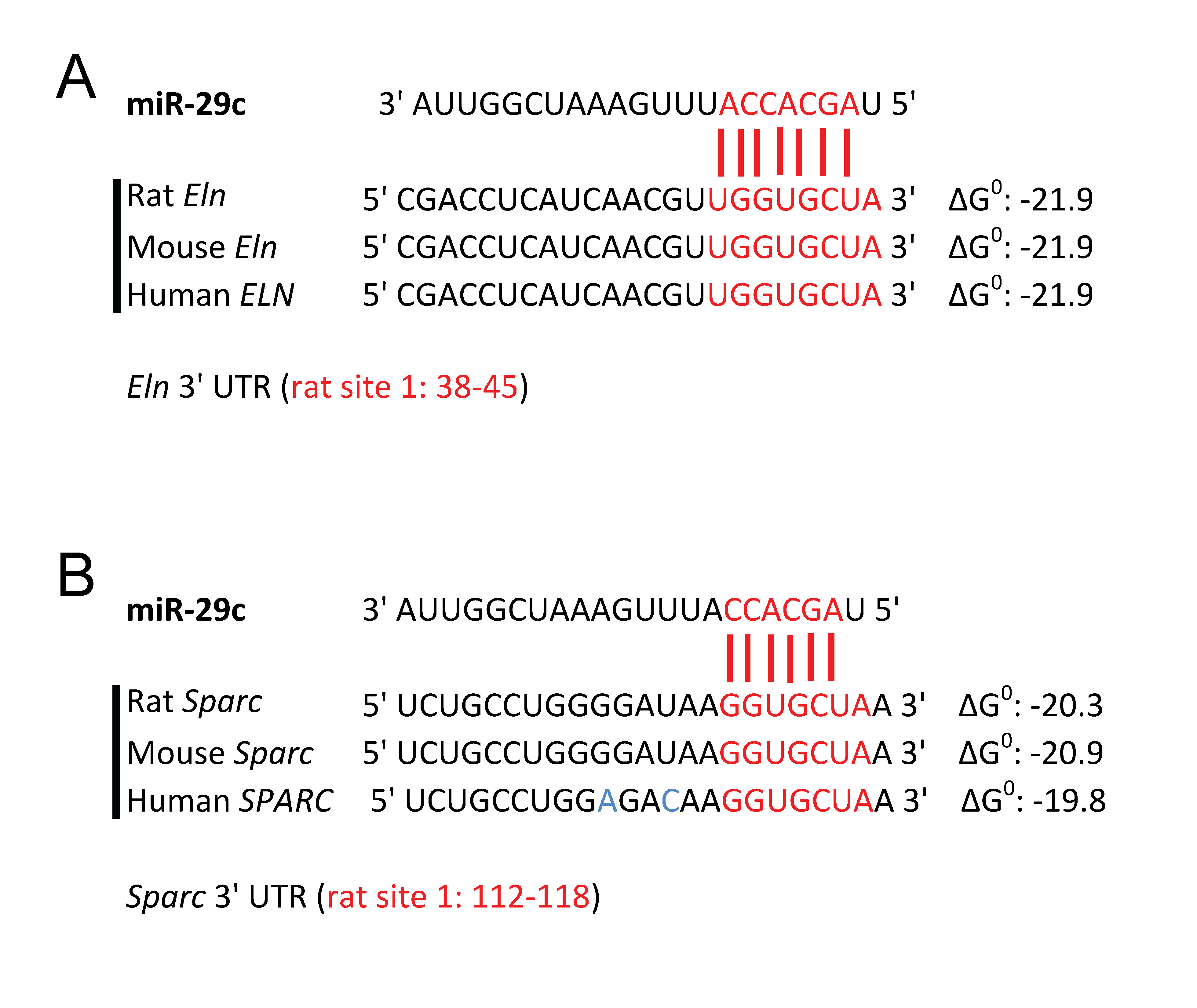

Supplement: Figure S1 — Free energies (ΔG0) of miR-29c binding to the proximal 3’UTR site in rat, mouse and human elastin (Eln, panel A) and osteonectin (Sparc, panel B), respectively. Base-pairing in the seed region is indicated by vertical lines. Additional nucleotides are also responsible for the binding strength. The free energies were obtained using the FindTar3 database. The binding sites shown, and the more distal sites, are all well conserved between species. (TIFF) [file pone.0082308.s001.tiff]

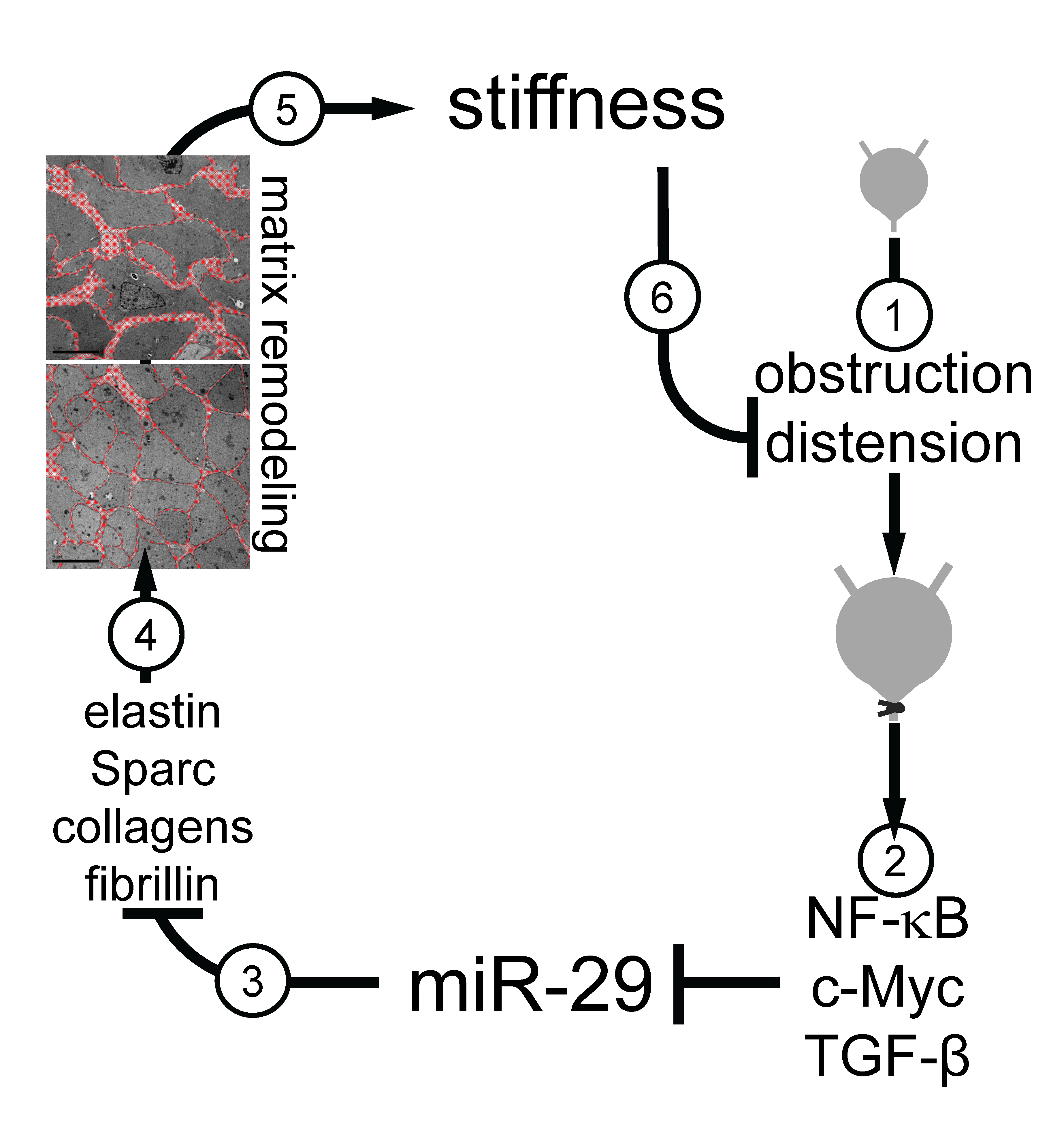

Supplement: Figure S2 — Flow chart showing the model proposed for miR-29 repression in outlet obstruction and for miR-29-mediated matrix remodeling and altered passive mechanical properties. Outlet obstruction such as seen in elderly men with enlarged prostate glands and in children with urethral valves leads to (1) distension of the detrusor. This in turn (2) activates multiple signaling pathways including c-Myc, NF-κB and TGF-β/SMAD3 that in turn repress miR-29. The reduced level of miR-29 leads to increased levels of mRNAs encoding extracellular matrix proteins (3), including elastin and Sparc (osteonectin), but possibly also collagens and fibrillin-1. The resulting protein synthesis and matrix deposition (4) leads to increased detrusor stiffness (5) (and increased elastic modulus) which counteracts (6) further distension. The proposed model fits the data presented in this article, but alternative interpretations are possible and steps upstream of miR-29 repression need in vivo corroboration. The electron micrographs were captured at a magnification of x6000 and are from control (bottom) and Dicer KO (top) detrusors. The extracellular matrix between muscle cells has been highlighted using transparent red color and scale bars represent 5 µm. (TIFF) [file pone.0082308.s002.tiff]
